# A case of ruptured mucinous cystadenoma of the pancreas with recurrence-free survival for 8 years

**DOI:** 10.1186/s40792-020-00816-x

**Published:** 2020-03-16

**Authors:** Atsuro Fujinaga, Teijiro Hirashita, Yukio Iwashita, Hiroaki Nakanuma, Kazuhiro Tada, Takashi Masuda, Yuichi Endo, Masayuki Ohta, Hideki Ono, Kazunari Murakami, Masafumi Inomata

**Affiliations:** 1 Department of Gastroenterological and Pediatric Surgery, Faculty of Medicine, Oita University, Idaigaoka 1-1, Hasama-machi, Oita, 879-5593 Japan; 2 Department of Gastroenterology, Oita Prefectural Hospital, Oita, Japan; 3 Department of Gastroenterology, Faculty of Medicine, Oita University, Oita, Japan

**Keywords:** Mucinous cystadenoma of the pancreas, MCA, Rupture

## Abstract

**Background:**

Pancreatic mucinous cystic neoplasm (MCM) presenting with rupture is extremely rare, and very few studies have followed up patients over the long term after ruptured mucinous cystadenoma (MCA). We report a case of ruptured MCA of the pancreas with recurrence-free survival for 8 years.

**Case presentation:**

A 28-year-old Japanese woman was admitted to the emergency department of a local hospital after experiencing acute abdominal pain. Abdominal computed tomography revealed massive ascites and the presence of a cystic tumor measuring 60 mm in diameter in the pancreatic tail. Conservative therapy with antibiotics and abdominal drainage were performed to treat peritonitis that occurred secondary to the ruptured pancreatic cystic tumor, after which the patient’s symptoms improved. The patient was referred to our department for further examination and treatment. We diagnosed a ruptured MCN and performed laparoscopic spleen-preserving distal pancreatectomy. Histopathological findings revealed ovarian-type stroma, which tested positive for estrogen and progesterone receptors by immunohistochemistry. The histopathological diagnosis was MCA. The postoperative course was uneventful, and the patient remains alive without any evidence of recurrence at 8 years postoperatively.

**Conclusion:**

A good prognosis is possible even in cases of ruptured MCA. Because of the risk of peritoneal dissemination after ruptured MCA, long-term follow-up is important.

## Background

Pancreatic mucinous cystic neoplasm (MCN) is characterized by mucin-producing columnar epithelium and ovarian-type stroma. MCN occurs almost exclusively in women aged 40–60 years. MCN is often localized to the pancreatic body or tail without affecting the pancreatic ducts [1]. Ruptured mucinous cystadenoma (MCA) is extremely rare, and very few studies have followed up patients over the long term after ruptured MCA. We report a case of ruptured MCA of the pancreas with recurrence-free survival for 8 years.

## Case presentation

**Fig. 1:**
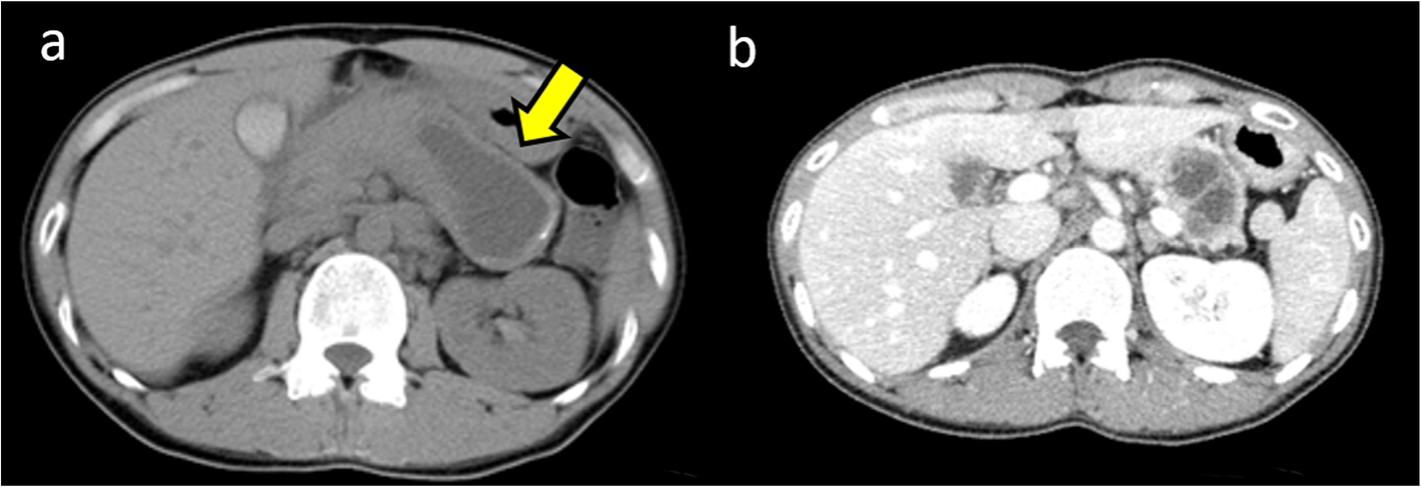
Computed tomography (CT) at onset revealed the presence of a cystic tumor measuring 60 mm in diameter in the pancreatic tail (**a**, arrow). Enhanced CT in our department also revealed a tumor in the pancreatic tail with an internal septal formation (**b**). There was no evidence of a nodular lesion in the tumor or ascites.

A 28-year-old Japanese woman was admitted to the emergency department of a local hospital after experiencing acute abdominal pain. Computed tomography (CT) imaging revealed massive ascites and the presence of a cystic tumor measuring 60 mm in diameter in the pancreatic tail (Fig. 1a). Conservative therapy with antibiotics and abdominal drainage were performed to treat peritonitis that occurred secondary to the ruptured pancreatic cystic tumor, after which the patient’s symptoms improved. Levels of amylase and carcinoembryonic antigen (CEA) in ascites were 3220 IU/L and 226.2 ng/mL, respectively, and cytological findings were negative for malignant findings. The patient was referred to our department for further examination and treatment. She had no previous medical history including pregnancy and no cigarette smoking or drinking. Her family history showed no remarkable findings.

**Fig. 2:**
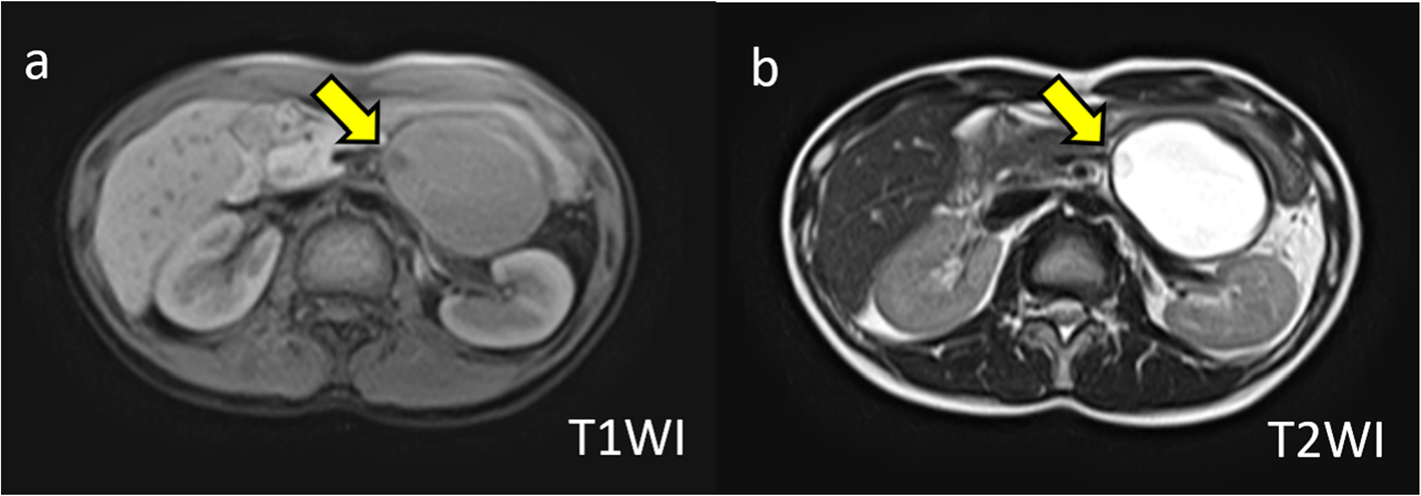
**a** T1-weighted image. A cystic tumor with an internal septal formation was present in the pancreatic tail. The cystic lesion showed a hypo-intense signal with nodular lesion (arrow). **b** T2-weighted image. The cystic lesion showed a hyperintense signal with nodular lesion (arrow)

**Fig. 3:**
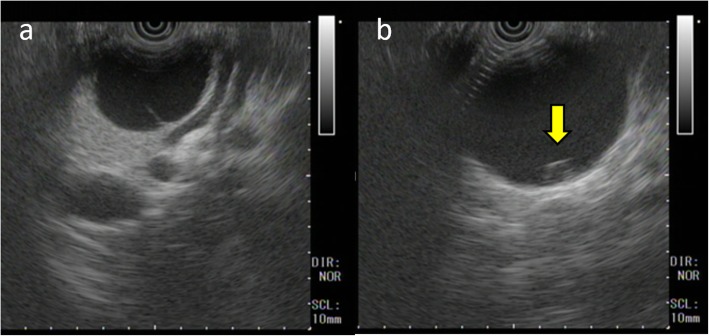
Endoscopic ultrasound revealed a multilocular cystic tumor (**a**) with nodular lesion (**b**, arrow).

On admission, her abdomen was flat and soft, and there was no abdominal pain. Laboratory tests showed no anemia, and liver and kidney functions were normal. Serum tumor markers CEA and CA19-9 were 0.8 ng/mL and 3620.9 U/mL, respectively. Enhanced CT imaging showed a tumor measuring 60 mm in diameter with an internal septal formation in the pancreatic tail (Fig. 1b). There was no evidence of nodular lesion in the tumor or ascites. Magnetic resonance imaging (MRI) showed a cystic tumor with an internal septal formation present in the pancreatic tail. T1- and T2-weighted imaging showed hypo- and hyperintense signals, respectively, with an unenhanced nodular lesion measuring 10 mm in diameter (Fig. 2a, b). Endoscopic ultrasound (EUS) also revealed a multilocular cystic tumor with nodular lesion (Fig. 3a, b). We diagnosed MCN after rupture. The tumor was close to the splenic vessels; therefore, we decided to perform a laparoscopic spleen-preserving distal pancreatectomy (SPDP) with splenic vascular resection (Warshaw’s technique).

**Fig. 4:**
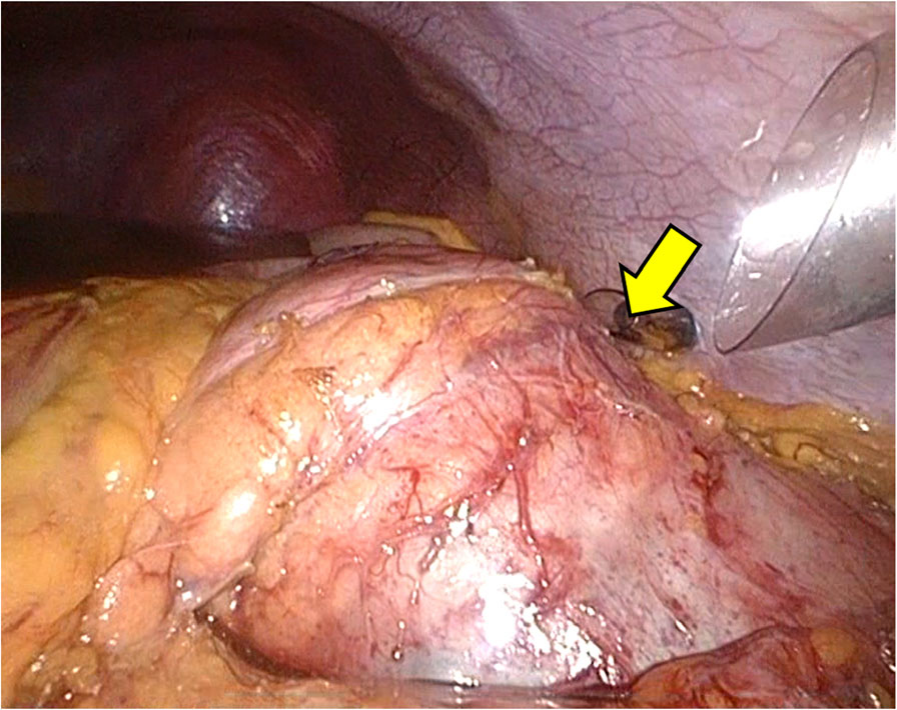
A soft tumor was identified in the pancreatic tail (arrow)

After induction of general anesthesia, the patient was positioned in the supine position. Complete exploration of the abdominal cavity revealed no ascites or peritoneal dissemination. The soft tumor was identified in the pancreatic tail (Fig. 4). To avoid rupturing the tumor, we performed fine needle aspiration (FNA) of the cystic fluid by using a balloon catheter. We aspirated 250 mL of yellow fluid and sutured the punctured tumor. Levels of AMY, CEA, and CA19-9 in the cystic fluid were 23,639 IU/L, 203.4 ng/mL, and 1,867,200 ng/mL, respectively. We cut the splenic artery and vein while preserving the left gastroepiploic and short gastric vessels. Then, we cut the pancreatic body with a linear stapler and removed the tumor while preserving the spleen. Finally, we confirmed blood flow to the spleen. The operative time was 339 min, and the blood loss was 80 mL. The specimen was a multilocular cystic tumor measuring 80 mm in diameter that contained mucus. Histopathological findings revealed columnar epithelium with mild dysplasia and ovarian-type stroma, which tested positive for estrogen receptor and progesterone receptor by immunohistochemistry. The histopathological diagnosis was MCA.

The patient remained in hospital for 10 days after surgery with no postoperative complications. The patient remains alive without any evidence of recurrence at 8 years postoperatively.

## Discussion

Ruptured MCN is extremely rare because of its thick fibrous capsule [2]. When we searched English articles in PubMed, seven cases of ruptured MCN including our case have been reported (Table 1) [3–8]. In these reported cases of ruptured MCN, the average patient age was 38 and all patients were women. Five cases were of mucinous cystadenocarcinoma (MCC), and two were of MCA. The average size was 116 mm, and the tumor was located in the pancreatic body or tail in six cases. The average follow-up period was 34 months and is longest in the present case. Local recurrence occurred only in one MCC case, and all patients survived during the follow-up period. Although the prognosis of MCN without malignancy is good after complete resection [1, 9], there is a possibility of peritoneal dissemination in ruptured MCN [10], and the long-term prognosis after rupture is unknown. In the present case, the patient remains alive without any evidence of recurrence at 8 years postoperatively. To our knowledge, this is the longest survival time among the reported cases of ruptured MCN.

**Table 1:** Reported cases of ruptured MCNs

	Authors (year)	Age (years)	Sex	Diagnosis	Tumor size (mm)	Location	Surgical procedure	Follow-up (months)	Recurrence	Outcome
1	Smithers et al. [3] (1986)	33	F	MCC	100	Body/tail	DP	Unknown	Unknown	Unknown
2	Ozden et al. [4] (2007)	32	F	MCC	150	Body/tail	SPDP	12	None	Alive
3	Bergenfeldt et al. [5] (2008)	42	F	MCC	200	Body	DP	19	None	Alive
4	Naganuma et al. [6] (2011)	32	F	MCC	110	Head	PD	36	Local recurrence	Alive
5	Imoto et al. [7] (2013)	69	F	MCC	60	Body/tail	DP	2	None	Alive
6	Haddad et al. [8] (2019)	30	F	MCA	111	Tail	DP	36	None	Alive
7	Our case	28	F	MCA	80	Tail	SPDP	96	None	Alive

*DP* distal pancreatectomy, *MCA* mucinous cystadenoma, *MCC* mucinous cystadenocarcinoma, *PD* pancreaticoduodenectomy, *SPDP* spleen-preserving distal pancreatectomy

WHO defines MCN as “A benign cystic tumour composed of columnar mucin-producing epithelium supported by ovarian-type stroma” [11]. The symptoms of MCN develop slowly, and non-specific gastrointestinal complaints or abdominal pain are common [5]. MCN occurs almost exclusively in women aged 40–60 years and is often localized to the pancreatic body or tail without affecting the pancreatic ducts [1]. In a report considering only cases with ovarian-type stroma, 98.1% were women with an average age of 48.1 years [9]. MCN was present in the pancreatic body or tail in 99.4% of the cases, and the average size was 65 mm [9]. In general, MCN does not communicate with the pancreatic ducts, but communication was observed in 18.1% of the cases [1, 9]. The general consensus is that when MCN is diagnosed, it should be promptly resected regardless of whether it is malignant because of its potential for malignancy [1, 12]. It is important not to injure the tumor during surgery [13]. Although EUS-FNA is useful for the diagnosis of pancreatic cystic tumors [14, 15], peritoneal dissemination has been reported in some cases after EUS-FNA [10]. Thus, the usefulness of EUS-FNA for pancreatic cystic tumors remains controversial, and it is not recommended in Japan [1]. However, EUS is considered the most useful modality for detecting nodular lesions in the cystic tumor [1, 16].

The cause of rupture has been reported to relate to the influence of female sex hormones on the behavior of MCN, especially during pregnancy [3, 4, 17]. Female sex hormones may affect estrogen and progesterone receptors of the MCN and promote the production of mucus [17]. The average size of a ruptured MCN is 116 mm, and it is larger than that of unruptured MCNs [9]. Therefore, the size of the MCN is thought to be related to rupture. As 5 of the 7 reported cases were women aged 20 to 40 years, female sex hormones, and not only those during pregnancy, might affect tumor growth and rupture.

Regarding prognosis after rupture, recurrence occurred in the cases of MCC, and a local pattern of recurrence was present. Although no recurrence was observed in the cases of MCA, the follow-up periods were short, and the long-term prognosis remains unclear. Therefore, there is little evidence regarding prognosis after a ruptured MCN. The 5-year survival rates after complete resection of unruptured MCN for adenoma and adenocarcinoma were 98.8% and 86.5%, respectively [9]. However, considering the malignant potential of MCN [1, 12] and the risk of peritoneal dissemination due the leakage of mucus [10, 18], long-term follow-up for at least 5 years on ultrasonography or CT is important to check for recurrence, including the appearance of ascites.

## Conclusions

A good prognosis might be expected in patients with MCA even in those with rupture. However, there is a risk of peritoneal dissemination with rupture, and long-term follow-up is thus considered important. To our knowledge, the present patient has survived without recurrence for the longest period among the reported cases of ruptured MCN.

## Data Availability

The data are not available for public access because of patient privacy concerns but are available from the corresponding author on reasonable request.

## Abbreviations

CT: Computed tomography
DP: Distal pancreatectomy
CEA: Carcinoembryonic antigen
EUS: Endoscopic ultrasound
EUS-FNA: Endoscopic ultrasound-fine needle aspiration
MCA: Mucinous cystadenoma
MCC: Mucinous cystadenocarcinoma
MCN: Mucinous cystic neoplasm
MRI: Magnetic resonance imaging
PD: Pancreaticoduodenectomy
SPDP: Spleen-preserving DP
